# Personalized Template-Based Step Detection From Inertial Measurement Units Signals in Multiple Sclerosis

**DOI:** 10.3389/fneur.2020.00261

**Published:** 2020-04-21

**Authors:** Aliénor Vienne-Jumeau, Laurent Oudre, Albane Moreau, Flavien Quijoux, Sébastien Edmond, Mélanie Dandrieux, Eva Legendre, Pierre Paul Vidal, Damien Ricard

**Affiliations:** ^1^COGNAC-G (UMR 8257), CNRS Service de Santé des Armées, University Paris Descartes, Paris, France; ^2^L2TI, University Paris 13, Villetaneuse, France; ^3^CMLA (UMR 8536), CNRS ENS Paris-Saclay, Cachan, France; ^4^Service de Neurologie, Hôpital d'Instruction des Armées Percy, Service de Santé des Armées, Clamart, France; ^5^ORPEA Group, Puteaux, France; ^6^Hangzhou Dianzi University, Zhejiang, China; ^7^École du Val-de-Grâce, Ecole de Santé des Armées, Paris, France

**Keywords:** multiple sclerosis, gait detection, gait quantification, gait disorders, wearable inertial sensors, inertial measurement unit, accelerometer

## Abstract

**Background:** Objective gait assessment is key for the follow-up of patients with progressive multiple sclerosis (pMS). Inertial measurement units (IMUs) provide reliable and yet easy quantitative gait assessment in routine clinical settings. However, to the best of our knowledge, no automated step-detection algorithm performs well in detecting severely altered pMS gait.

**Method:** This article elaborates on a step-detection method based on personalized templates tested against a gold standard. Twenty-two individuals with pMS and 10 young healthy subjects (HSs) were instructed to walk on an electronic walkway wearing synchronized IMUs. Templates were derived from the IMU signals by using Initial and Final Contact times given by the walkway. These were used to detect steps from other gait trials of the same individual (intra-individual template-based detection, IITD) or another participant from the same group (pMS or HS) (intra-group template-based detection, IGTD). All participants were seen twice with a 6-month interval, with two measurements performed at each visit. Performance and accuracy metrics were computed, along with a similarity index (SId), which was computed as the mean distance between detected steps and their respective closest template.

**Results:** For HS participants, both the IITD and the IGTD algorithms had precision and recall of 1.00 for detecting steps. For pMS participants, precision and recall ranged from 0.94 to 1.00 for IITD and 0.85 to 0.95 for IGTD depending on the level of disability. The SId was correlated with performance and the accuracy of the result. An SId threshold of 0.957 (IITD) and 0.963 (IGTD) could rule out decreased performance (F-measure ≤ 0.95), with negative predictive values of 0.99 and 0.96 with the IITD and IGTD algorithms. Also, the SId computed with the IITD and IGTD algorithms could distinguish individuals showing changes at 6-month follow-up.

**Conclusion:** This personalized step-detection method has high performance for detecting steps in pMS individuals with severely altered gait. The algorithm can be self-evaluating with the SI, which gives a measure of the confidence the clinician can have in the detection. What is more, the SId can be used as a biomarker of change in disease severity occurring between the two measurement times.

## 1. Introduction

Multiple sclerosis (MS) is a demyelinating disease of the central nervous system with varying clinical presentation and progression. Gait impairment is a hallmark of MS, and lower-limb function is perceived as the most important bodily disability across the disease spectrum (1). Thus, objective gait assessment is needed in both routine clinical care and clinical research trials to improve gait and balance follow-up in people with MS. Stopwatch-timed tests and clinical scales are used in routine daily practice to assess gait impairments. However, they are prone to practice effects (2) and variability (3, 4). We need objective and easy-to-perform gait assessment tools to detect such alterations.

Wearable inertial measurement units (IMUs) are such tools. They can outperform the classification accuracy of clinical tests for the risk of falling (5, 6) and can help in the follow-up of treatment efficacy (7, 8). Moreover, they are light, inexpensive, non-invasive and easy to use, which potentiates their implementation in clinical settings (8–11). To allow for routine use of IMUs, algorithms for automated step detection and computation of gait features of interest have been developed (12). However, few algorithms have been validated in individuals with progressive MS (pMS) (13), and individuals with severe disease are often not served well (14, 15). Techniques for step detection mostly rely on the use of filtering, thresholding, zero-crossing, or peak detection techniques (16–18) that are applied on the accelerometer/gyroscope signals. They rely on the hypothesis that steps are defined by characteristic events that can be isolated from the signals after preprocessing is applied to highlight these given events. These methods depend on the tuning of several factors (width of the bandpass filter, thresholds, etc.), which are often set empirically. Early works on the topic often used single IMUs and failed to adapt to different types of cohorts (19). More recent work with bilateral lower limb sensors has provided promising results for moderate to severe conditions but is still rare in the literature (12, 20). For these reasons, the use of templates or several techniques based on machine learning (18, 21) or Dynamic Time Warping (22–24) has been advocated in several articles (25, 26) as a way to automatically learn the characteristics of a cohort. However, heavily altered steps might not be caught by these templates (14, 25, 27). In particular, to the best of our knowledge, no automated step-detection method has been found sufficiently robust to be used for individuals with severe MS (Expanded Disability Status Scale [EDSS] 4 to 6).

In that context, we present a step-detection algorithm for detecting the steps of individuals with pMS that is based on their own walk. To abstract from calibration, we based our algorithms on templates and used those directly obtained from the individuals themselves. In this article, we describe how this personalized template-based algorithm method performs in detecting subsequent steps by the individual. We used the GaitRite® system as the gold standard to recognize gait events via the IMU signals. We derived a measure of similarity between templates and detected steps, the similarity index (SId) (28), and we show that this measure is a marker of the performance and accuracy of the algorithm, thus providing the clinician with an indicator of confidence in detecting steps of individuals with pMS.

## 2. Materials and Methods

### 2.1. Participants

We enrolled 22 individuals with pMS and 10 young healthy subjects (HSs) in this longitudinal prospective study. pMS individuals were consecutively recruited from the outpatient clinic of Percy Hospital (Clamart, France) between June 2018 and September 2018. HS participants were recruited from the hospital and research unit staff between June 2018 and September 2018. The inclusion criteria for the pMS cohort was age ≥ 18 years, a diagnosis of primary progressive or secondary progressive MS according to the 2010 International Panel criteria (29), ability to walk two sets of 6 m forward and then back with a U-turn, and no other conditions that could be a cause of altered gait. Pregnancy was an exclusion criterion. The inclusion criteria for the HS cohort were age 18–30 years, no report of falls in the past 5 years before inclusion [falls being defined as events that lead the standing or walking subject to a lower level on the ground unintentionally, without being externally pushed or pulled, regardless of whether an injury is sustained (30)], and no disease that could affect walking. The pMS and HS cohorts are not matched in age since the aim of the present manuscript is not to compare these two populations but rather to investigate the performances of the proposed method at both extremities of gait quality.

All pMS and HS participants provided written informed consent before inclusion. The study protocol followed the principles of the Declaration of Helsinki. It was approved by the ethics committee Protection des Personnes Nord Ouest III (ID RCB: 2017-A01538-45).

### 2.2. Gait Measurement Protocol

Two XSens® sensors (XSens® Technologies, Enschede, the Netherlands)—hereafter XS—were placed on the participant's body (one on the dorsal part of each foot) by using Velcro bands. The GaitRite®—hereafter GR—was the gold-standard. The data were sampled at 100 Hz for the XS and at 120 Hz for the GR. Both systems were synchronized in time by using the PC clock connected to the XS sensors. Participants performed two walks of 12 m with a U-turn (6 m on the way in and 6 m on the way out) at the first visit (month 0 [M0]) and again at the second visit, 6 months later (M6).

### 2.3. Algorithm Procedure

**Figure 1:**
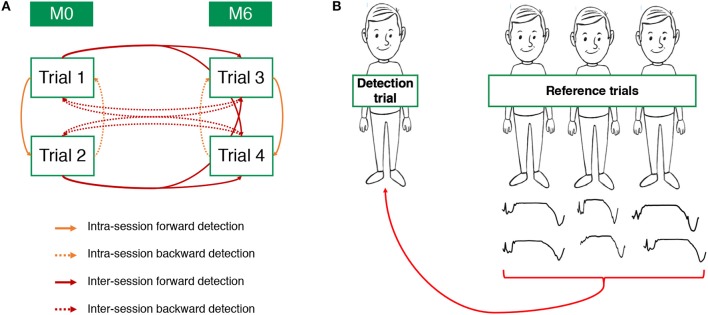
Definition of the different pairs of reference/detection trials analyzed by the intra-individual template-based detection (IITD) algorithm **(A)** and the intra-group template-based detection (IGTD) algorithm **(B)**.

The step-detection algorithms used for this study are based on previously published algorithms by Oudre et al. (25) and Vienne-Jumeau et al. (28). The processing is composed of three parts:

1. *Construction of the library of templates from the reference trial(s)*. The GR annotations are used to estimate the Initial Contacts (ICs) and Final Contacts (FCs). These times are then reported on the synchronized XS signals so as to extract waveforms corresponding to step templates (see Figure 1B). These templates are then stored in a library of templates.
2. *Step detection on the detection trial*. This library of templates is then used to detect the steps in the XS signals of the detection trial. The detection is performed through a template-matching technique involving an iterative greedy procedure [see (25) for details].
3. *Computation of the SId score*. When templates from the reference trial(s) are used to detect steps from the detection trial, it is possible to assess whether the templates of the library are *close* to those present in the signal. This proximity is quantified by the similarity index (SId) introduced in Vienne-Jumeau et al. (28), which is a score between 0 and 1 that reflects the similarity between the reference trial(s) and the detection trial.

The versatility of the algorithm allows the investigation of several configurations, whether the reference and detection trials belong to the same subject or not. In this article, we present two main configurations: intra-individual template-based detection (IITD) where the reference and detection trials belong to the same subject and intra-group template-based detection (IGTD) where they belong to different subjects.

#### Intra-individual template-based detection (IITD)

For this configuration, the reference and detection trials belong to the same subject. Since each subject has performed four different trials, it is possible to compare each trial with each of the others. For the sake of clarity, we classified these comparisons into four IITD categories (Figure 1A).

- Intra-session forward detection: the detection trial was performed during the same visit as the reference trial, and detection was after this trial. Pairs of reference/detection trials are trial 1/trial 2 and trial 3/trial 4.
- Intra-session backward detection: the detection trial was performed during the same visit as the reference trial, and detection was before this trial. Pairs of reference/detection trials are trial 2/trial 1 and trial 4/trial 3.
- Inter-session forward detection: the detection trial was performed at M6, and the reference trial was performed at M0. Pairs of reference/detection trials are trial 1/trial 3, trial 2/trial 3, trial 1/trial 4, and trial 2/trial 4.
- Inter-session backward detection: the detection trial was performed at M0, and the reference trial was performed at M6. Pairs of reference/detection trials are trial 3/trial 1, trial 3/trial 2, trial 4/trial 1, and trial 4/trial 2.

#### Intra-group template-based detection algorithm (IGTD)

For this configuration, the reference and detection trials belong to different subjects. However, we here only compare subjects belonging to the same group (pMS or HS), hence the *intra-group* term. First, we use the reference trials to extract for each subject the two more representative steps, i.e., the steps that are the most correlated with all steps from the subject. Then, we remove from this library the two steps belonging to the subject involved in the detection trial and perform the detection (see Figure 1B). Note that since the pMS and HS group do not have the same number of subjects, the library contains 42 templates for the pMS group and 18 for the HS group.

### 2.4. Performance and Accuracy Metric Definitions

ICs and FCs can be used to compute several gait parameters such as step, stride, stance, and swing times (31). A step is defined as the period between two successive ICs of opposite feet, while a stride corresponding to the period between two successive ICs of the same foot. The stance phase is defined as the period between the IC and FC of the same foot, while the swing phase corresponds to the period between the FC and the next IC of the opposite foot. In this article, we chose to only output the error made in the estimation of the IC, FC, and the stance phase durations.

#### 2.4.1. Performance

The performances of the detection algorithms (IITD and IGTD) are assessed by comparing the detected times for IC and FC to those output by the GR gold standard. The following metrics were used:

- Precision (also called positive predictive value). The precision is the number of true detected steps divided by the total number of detected steps.
- Recall (also called sensitivity). The recall is the number of true detected steps divided by the total number of true steps.
- F-measure (also called F1 score). The F measure is the harmonic mean of precision and recall, thus defined as:
  (1) $$\begin{array}{l} Fmeasure=\frac{2\times Precision\times Recall}{Precision+Recall} \end{array}$$

#### 2.4.2. Accuracy

The following metrics are computed for each true detected step:

- ΔIC: absolute difference between the detected and the true ICs
  $$\begin{array}{l} \Delta \text{IC}=|{\text{IC}}_{detected}-{\text{IC}}_{GaitRite}| \end{array}$$
- ΔFC: absolute difference between the detected and the true FCs
  $$\begin{array}{l} \Delta \text{FC}=|{\text{FC}}_{detected}-{\text{FC}}_{GaitRite}| \end{array}$$
- ΔStance: absolute difference between the detected and the true stance durations
  $$\begin{array}{l} \Delta \text{Stance}=|\left({\text{FC}}_{detected}-{\text{IC}}_{detected}\right)-\left({\text{FC}}_{GaitRite}-{\text{IC}}_{GaitRite}\right)| \end{array}$$

### 2.5. Clinically Meaningful Change

The SId was evaluated as a predictor of change in a disease state. To that end, the thresholds for modification in states were defined on the basis of an evidence-based clinically meaningful change in values for conventional scales as retrieved from the literature. Four common scales were evaluated: the Expanded Disability Status Scale (EDSS) with underlying Functional Status (FS) scores (32), the Multiple Sclerosis Walking Scale-12 (MSWS) (18), and the Fatigue Impact Scale (FIS) (33, 34).

Meaningful changes in values on these scales were defined according to the literature. For the EDSS and the FS scores, any change (from a 0.5-point change) was considered meaningful (32). A significant change of 20 was chosen for the MSWS because the minimal important difference was found to be between 20 and 22 (depending on the use of a walking aid) (35). For FIS, the threshold of significant change was 20 (36). A change in any of these domains was considered as a change in disease state.

### 2.6. Statistical Analysis

All parameters were tested for normality with Shapiro–Wilks tests. Data are reported with means and standard deviations (SDs) as well as medians and interquartile ranges (IQRs) for non-normally distributed data.

#### 2.6.1. Performance and Accuracy

Performance and accuracy were compared between the two participant groups by using the Student's *t*-test for data with normal distribution and the Mann–Whitney *U*-test for data with non-normal distribution. An F-measure ≤ 0.95 was considered low performance.

#### 2.6.2. Similarity Index

To evaluate SId as a predictor of low performance (F-measure ≤ 0.95), we performed Monte Carlo cross-validation nested by four-fold cross-validation (37). Two thirds (15/22) of the pMS individuals were included in the training cohort and the remaining one third (7/22) in the test, or validation, cohort. Receiver operating characteristic (ROC) curves were computed for the IITD and IGTD algorithms. Discrimination was assessed in the test cohort by estimating negative and positive predictive values, sensitivity, specificity, and the area under the ROC curve (AUC). The best threshold was determined by two distinct methods. First, the cut-point that optimizes the test's differentiating ability with equal weight given to sensitivity and specificity, called the Youden index (Y) (38), was used as recommended by Perkins and Schisterman (39). Second, a conditional Y (*Y*_*c*_), defined as a Youden index Y with the constraint that sensitivity be > 90% was also defined:

(2) $$\begin{array}{l} Y_{c}=\underset{sensitivity>90}{\max}sensitivity+specificity-1 \end{array}$$

Sensitivities, specificities, Y, *Y*_*c*_, and the AUC were computed on 1,000 configurations of the training and test cohorts and are reported as means with corresponding 95% confidence intervals (CIs). The average ROC curves were used to estimate negative and positive predictive values by using the mean sensitivities and specificities.

To evaluate the SId as a predictor of clinical state, Pearson correlation coefficients were computed between the SId and EDSS, MSWS, and FIS. If correlation with the EDSS was significant, further correlations with functional scores potentially affecting walking (pyramidal, cerebellar, bulbar, sensitive, and cognitive scores) were computed.

To evaluate the SId as a predictor of change in clinical severity, two groups of patients were constituted depending on whether their severity changed in at least one domain (EDSS, EDSS FS, MSWS, or FIS) between M0 and M6. SId was compared between these two groups (see Section 2.5).

Primary data analysis involved using MATLAB® R2019a. Statistical analysis involved using R v3.5.1. Correction for multiple comparisons using Bonferroni adjustment was applied to all tests.

## 3. Results

### 3.1. Participants

We included 22 pMS individuals and 10 HS individuals. The baseline characteristics of the participants are reported in Table 1. pMS individuals were divided into two groups depending on their level of disability: usually needing a walking aid for walking short distances (EDSS ≥ 6.0) (WA-pMS group: pMS and needing a walking aid) and needing a walking aid neither usually nor during the test (NA-pMS group: pMS not needing a walking aid). Mean (SD) age was 57 (9), 59 (13), and 26 (1) for the WA-pMS, NA-pMS, and HS groups, respectively. For the WA-pMS group, the median EDSS was 6.0 [IQR 6.0–6.5] and for the NA-pMS group, 3.5 [IQR 3.0–5.0]. Two individuals from the WA-pMS group did not use a walking aid during the test (but required a person to follow them closely). The gait parameters output by the GaitRite are displayed in Table 2. A list of subjects with clinically meaningful change and their characteristics is displayed in Table 3.

**Table 1:** Baseline characteristics of participants from the three groups: progressive multiple sclerosis (pMS) and needing a walking aid (WA-pMS) and not needing a walking aid (NA-pMS) and healthy subjects (HS).

	**WA-pMS** **(*n* = 9)**	**NA-pMS** **(*n* = 13)**	**HS** **(*n* = 10)**
Sex (male/female)	4 / 5	5 / 8	4 / 6
Age (years)	57 (9)	59 (13)	26 (1)
Height (m)	1.69 (0.08)	1.72 (0.10)	1.72 (0.09)
Weight (kg)	62.3 (17.8)	77.3 (13.1)	58.2 (10.9)
Body mass index (kg/m^2^)	21.6 (4.2)	26.2 (4.9)	21.0 (3.0)
EDSS	6.0 [6.0–6.5]	3.5 [3.0–5.0]	-
EDSS - pyramidal	4.0 [3.0–4.0]	3.0 [2.0–3.0]	-
EDSS - cerebellar	2.0 [1.0–3.0]	1.0 [0.0–2.0]	-
EDSS - bulbar	0 [0.0–2.0]	0.0 [0.0–0.0]	-
EDSS - sensitive	2.0 [1.0–2.0]	2.0 [1.0–2.0]	-
EDSS - cognitive	2.0 [1.0–3.0]	0.0 [0.0–1.0]	-
MSWS	66 (19)	64 (17)	-
FIS	39 (23)	46 (27)	-
Walking aid during the test (/total number)	7 / 9	0 / 13	0 / 10
Unilateral cane	1	0	-
Bilateral cane	3	0	-
Walker	1	0	-
Human help	1	0	-
Cane + human help	1	0	-

*Data are mean (SD) or median (IQR) EDSS, Expanded Diseases Status Scale; MSWS, Multiple Sclerosis Walking Scale; FIS, Fatigue Impact Scale*.

**Table 2:** Number of steps and mean (sd) of gait velocities, step time, step length, and double stance time for all groups.

	**WA-pMS (*****n*** **=** **9)**		**NA-pMS (*****n*** **=** **13)**		**HS (*****n*** **=** **10)**	
	**M0**	**M6**	**M0**	**M6**	**M0**	**M6**
Total number of steps	547	509	383	396	440	448
Gait velocity (m/s)	0.42 (0.16)	0.41 (0.13)	0.77 (0.17)	0.81 (0.18)	1.18 (0.12)	1.18 (0.14)
Step time (s)	0.79 (0.15)	0.80 (0.20)	0.65 (0.08)	0.63 (0.07)	0.51 (0.03)	0.51 (0.04)
Step length (m)	0.34 (0.15)	0.34 (0.12)	0.52 (0.10)	0.51 (0.10)	0.49 (0.03)	0.49 (0.03)
Double stance time (% of stride duration)	45 (8)	45 (9)	33 (5)	33 (5)	22 (3)	22 (3)

*All values are provided by the gold standard GaitRite*.

**Table 3:** List of the eight subjects with clinically meaningful changes between M0 and M6.

**Group**	**EDSS**	**MSWS**	**FIS**
WA-pMS	6.5 / 6	83 / 93	56 / 70
WA-pMS	6.5 / 6.5	83 / 95	33 / 68
WA-pMS	6 / 6	20 / 62	0 / 5
WA-pMS	5.5 / 6	78 / 62	42 / 55
WA-pMS	6 / 6	63 / 58	58 / 70
NA-pMS	5 / 4.5	58 / 75	55 / 55
NA-pMS	3.5 / 3.5	88 / 57	55 / 30
NA-pMS	2.5 / 3.5	63 / 82	62 / 78

*EDSS, MSWS, and FIS are displayed at M0 / M6*.

### 3.2. Performance and Accuracy of the Detection Method

#### 3.2.1. Performance

**Figure 2:**
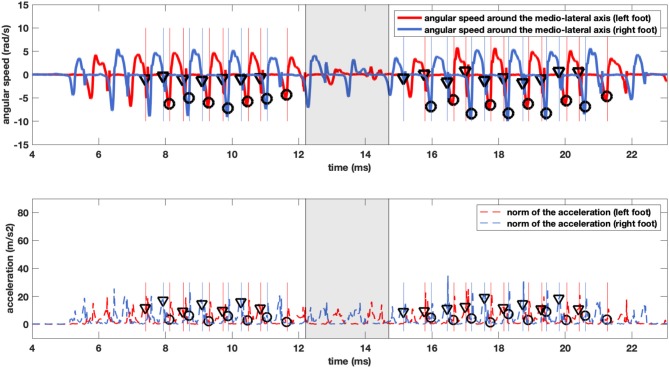
Example traces of the results of the step detection method for a pMS individual. The lines represent medio-lateral axis angular velocity (upper panel) and the magnitude of the norm of the acceleration (lower panel) recorded from the right (blue) and left (red) feet. The vertical lines display the Initial and Final Contacts as defined by the GR, and the triangles and circles display the ICs (triangles) and FCs (circles) as detected by our method. The shaded zone delimits the U-turn and is excluded from the analysis.

Figure 2 shows an example of a pair of reference and detection trials with gait events detected by using the IITD algorithm. Performance scores with the IITD method are given in Table 4, with *p*-values comparing the two groups of pMS participants.

**Table 4:** Performance (precision, recall, and F-measure) scores for the intra-individual template-based detection (IITD) algorithm.

		**WA-pMS** **(*n* = 9)**	**NA-pMS** **(*n* = 13)**	**HS** **(*n* = 10)**	*p*-value^*^
Forward intra-session	Precision	0.98 (0.03)	1.00 (0.01)	1.00 (0.00)	0.04
	Recall	1.00 (0.01)	1.00 (0.01)	1.00 (0.00)	0.62
	F-measure	0.99 (0.02)	1.00 (0.01)	1.00 (0.00)	0.05
Backward intra-session	Precision	0.97 (0.06)	1.00 (0.02)	1.00 (0.00)	0.21
	Recall	0.96 (0.12)	1.00 (0.01)	1.00 (0.00)	0.27
	F-measure	0.97 (0.10)	1.00 (0.01)	1.00 (0.00)	0.24
Forward inter-session	Precision	0.99 (0.03)	0.99 (0.02)	1.00 (0.00)	0.14
	Recall	0.99 (0.02)	0.99 (0.03)	1.00 (0.00)	0.58
	F-measure	0.99 (0.02)	0.99 (0.02)	1.00 (0.00)	0.38
Backward inter-session	Precision	0.98 (0.03)	0.99 (0.05)	1.00 (0.00)	0.91
	Recall	0.94 (0.17)	0.99 (0.05)	1.00 (0.00)	0.13
	F-measure	0.95 (0.14)	0.99 (0.04)	1.00 (0.00)	0.18

*Data are mean (SD)*.

* *Comparing WA-pMS and NA-pMS participants*.

For HS individuals, the IITD algorithm showed precision and recall of 1.00 for all detections (intra-session and inter-session, forward and backward). WA-pMS and NA-pMS individuals had equal performance (*p* > 0.05 for all configurations), with precision and recall from 0.94 (SD 0.17) to 1.00 (SD 0.01). The difference between forward and backward prediction was not significant for any of the three measures of performance (*p* > 0.05 for intra- and inter-session for all three measures).

The performance scores for the IGTD algorithm are given in Table 5. Precision and recall again reach 1.00 for HS participants. Precision and recall were 0.85 (SD 0.21) and 0.93 (SD 0.15), respectively, for the WA-pMS group and were 0.92 (SD 0.19) and 0.95 (0.17) for the NA-pMS group.

**Table 5:** Performance (precision, recall, and F-measure) scores for the intra-group template-based detection (IGTD) algorithm.

	**WA-pMS** **(*n* = 9)**	**NA-pMS** **(*n* = 13)**	**HS** **(*n* = 10)**	*p*-value^*^
Precision	0.85 (0.21)	0.92 (0.19)	1.00 (0.00)	0.12
Recall	0.93 (0.15)	0.95 (0.17)	1.00 (0.00)	0.44
F-measure	0.88 (0.18)	0.93 (0.18)	1.00 (0.00)	0.16

*Data are mean (SD)*.

* *Comparing WA-pMS and NA-pMS participants*.

The performances of the IITD (inter-session) and IGTD algorithms did not differ for the HS group (precision and recall of 1.00). Both the NA-pMS and WA-pMS groups showed altered precision with the IGTD vs. the IITD (*p* = 0.001 for WA-pMS, and 0.009 for NA-pMS). Recall was not decreased (*p* = 0.21 for WA-pMS, and 0.11 for NA-pMS). The resulting F-measures differed between the two algorithms (*p* = 0.011 for WA-pMS, and 0.023 for NA-pMS).

#### 3.2.2. Accuracy

The accuracy scores with the IITD algorithm are given in Table 6. For all configurations, the mean time difference in stance duration between the detection and the gold standard was lower than 0.14 s (SD 0.08) for the WA-pMS group and lower than 0.02 s (SD 0.02) for HS participants. These stance duration errors approximately correspond to 1.8% of the total stride duration for HS subjects, 3.3% for NA-pMS patients, and 9.6% for WA-pMS patients.

**Table 6:** Accuracy (ΔIC, ΔFC, and ΔStance) scores for the IITD algorithm.

		**WA-pMS** **(*n* = 9)**	**NA-pMS** **(*n* = 13)**	**HS** **(*n* = 10)**	*p*-value^*^
Forward intra-session	ΔIC (s)	0.08 (0.08)	0.03 (0.05)	0.01 (0)	**0.030**
	ΔFC (s)	0.05 (0.07)	0.02 (0.02)	0.01 (0.01)	0.136
	ΔStance (s)	0.1 (0.07)	0.04 (0.06)	0.01 (0.01)	**0.020**
Backward intra-session	ΔIC (s)	0.09 (0.11)	0.03 (0.03)	0.01 (0.01)	0.058
	ΔFC (s)	0.06 (0.11)	0.02 (0.02)	0.02 (0.01)	0.129
	ΔStance (s)	0.09 (0.07)	0.04 (0.05)	0.01 (0.01)	**0.009**
Forward inter-session	ΔIC (s)	0.1 (0.06)	0.06 (0.07)	0.02 (0.01)	**0.014**
	ΔFC (s)	0.05 (0.04)	0.03 (0.04)	0.02 (0.01)	0.054
	ΔStance (s)	0.13 (0.07)	0.07 (0.09)	0.02 (0.01)	**0.002**
Backward inter-session	ΔIC (s)	0.1 (0.07)	0.05 (0.05)	0.01 (0.01)	** <0.0001**
	ΔFC (s)	0.07 (0.09)	0.03 (0.02)	0.02 (0.01)	**0.024**
	ΔStance (s)	0.14 (0.08)	0.06 (0.07)	0.02 (0.01)	** <0.0001**

*Data are mean (SD)*.

* *Comparing WA-pMS and NA-pMS participants*.

*P-values lower than 0.05 are displayed in bold*.

The accuracy scores with the IGTD algorithm are given in Table 7. The mean time difference in Stance duration for HS participants was 0.03 s (SD 0.02), with equal shifts in ICs and FCs [0.02 s (SD 0.01)]. The mean time difference was high for both the NA-pMS and WA-pMS groups, with a predominant shift in ICs vs. FCs [mean IC difference of 0.19 s (SD 0.11) and 0.10s (SD 0.15) for the WA-pMS and NA-pMS groups]. These stance duration errors approximately correspond to 2.8% of the total stride duration for HS subjects, 5.4% for NA-pMS patients, and 18.2% for WA-pMS patients.

**Table 7:** Accuracy (ΔIC, ΔFC, and ΔStance) scores for the IGTD algorithm.

	**WA-pMS** **(*n* = 9)**	**NA-pMS** **(*n* = 13)**	**HS** **(*n* = 10)**	*p*-value^*^
ΔIC (s)	0.19 (0.11)	0.10 (0.15)	0.02 (0.01)	0.002
ΔFC (s)	0.09 (0.11)	0.03 (0.04)	0.02 (0.01)	0.003
ΔStance (s)	0.27 (0.17)	0.12 (0.19)	0.03 (0.02)	<0.0001

*Data are mean (SD)*.

* *Comparing WA-pMS and NA-pMS participants*.

Comparisons between the IITD (inter-session) and the IGTD algorithms showed significant differences for ΔIC (*p*-value: WA-pMS: 0.0001; NA-pMS: 0.028; HS: 0.027) and ΔStance (*p*-value: WA-pMS: 0.13; NA-pMS: 0.84; HS: 0.65) but not for ΔFC (*p*-value: WA-pMS: < .0001; NA-pMS: 0.038; HS: 0.007).

### 3.3. Self-Evaluation Using the SId

**Figure 3:**
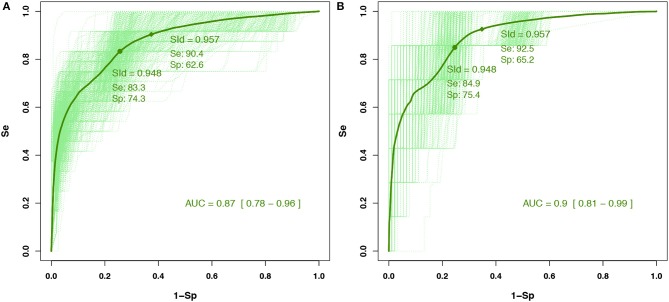
Receiver operating characteristic (ROC) curves for **(A)** the training cohort involving 15 people with pMS and **(B)** the test cohort involving seven people with pMS for the IITD detection method. Cutoffs were determined with the training cohort, and their predictive values were computed within the test cohort. Dashed curves are ROC curves for each configuration of the Monte Carlo cross-validation (and the nested four-fold cross-validation for the training set). Plain curves are the means of all dashed curves.

Through analyzing the ROC curves with the IITD (intra- or inter-sessions), it was found that an SId cutoff of 0.93 had the best Y (accuracy) for detecting an F-measure ≤ 0.95 in the training cohort (*n* = 15) (Figure 3A). The prevalence of decreased performance (F-measure ≤ 0.95) was 7% with the IITD method. An SId cutoff of 0.94 had the best *Y*_*c*_ (accuracy with constraint on sensitivity to bias the test toward screening). In a validation study, among the pMS testing cohort (*n* = 7) (Figure 3B), an SId of ≤ 0.94 had a negative predictive value (i.e., F-measure above 0.95) of 99.2% (95% Confidence Interval (CI): 99.1–99.3%), with 92.5% sensitivity (95% CI 91.8–93.3%) and 65.2% specificity (95% CI 64.4–66.0%) The AUC with the testing cohort was 0.90 (95% CI 0.81–0.99).

**Figure 4:**
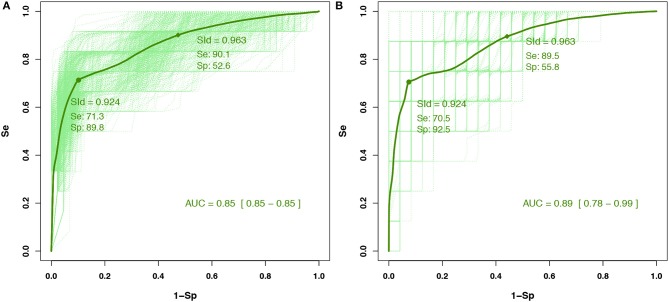
Receiver operating characteristic (ROC) curves for **(A)** the training cohort involving 15 people with pMS and **(B)** the test cohort involving seven people with pMS for the IGTD detection method. Cutoffs were determined with the training cohort, and their predictive values were computed within the test cohort. Dashed curves are ROC curves for each configuration of the Monte Carlo cross-validation (and the nested four-fold cross-validation for the training set). Plain curves are the means of all dashed curves.

The prevalence of decreased performance (F-measure ≤ 0.95) was 22% with the IGTD method. On analysis of ROC curves for IGTD, an SId cutoff of 0.92 has the best Y (accuracy) for detecting an F-measure ≤ 0.95 in the training cohort (*n* = 15) (Figure 4A). An SId cutoff of 0.96 had the best *Y*_*c*_ (accuracy with constraint on sensitivity to bias the test toward screening). In a validation study, for the pMS testing cohort (*n* = 7) (Figure 4B), an SId of ≤ 0.96 had a negative predictive value (i.e., F-measure above 0.95) of 96.0% (95% CI 95.7–96.3%), with 89.5% sensitivity (95% CI 88.5–90.5%) and 55.8% specificity (95% CI 54.8–56.9%). The AUC with the testing cohort was 0.89 (95% CI 0.78–0.99).

### 3.4. Correlation of the SId With Disease Severity

The correlations between SId and disease severity scores are displayed on Table 8. The SId computed with the IITD algorithm for the whole pMS cohort showed a low correlation with the EDSS (Pearson *r*: −0.38; *p* : < .0001) and its pyramidal and sensitivity functional subscores (pyramidal FS: Pearson *r*: −0.32; *p*: < .0001 and sensitivity FS: *r*: −0.22; *p*: 0.008) but not the other subscores. The SId did not show correlation with either the MSWS (*p*: 0.058) or the FIS (*p*: 0.647).

**Table 8:** Pearson correlations of severity scores with the IITD and IGTD algorithms for pMS participants (*n* = 22).

	**IITD**		**IGTD**	
	***r***	***p*-value**	***r***	***p*-value**
EDSS	-0.38	** <0.0001**	-0.15	0.585
EDSS - pyramidal	-0.32	** <0.0001**	-	-
EDSS - cerebellar	-0.01	0.899	-	-
EDSS - bulbar	-0.17	**0.044**	-	-
EDSS - sensitive	-0.22	**0.008**	-	-
EDSS - cognitive	-0.11	0.168	-	-
MSWS	-0.15	0.059	-0.39	0.070
FIS	0.04	0.647	-0.2	0.376

*P-values lower than 0.05 are displayed in “bold”*.

The SId computed with the IGTD for the whole pMS cohort was not correlated with the EDSS (*p*: 0.585), the MSWS, or the FIS.

### 3.5. Change in Clinical State Reflected in the IITD Similarity Index

**Figure 5:**
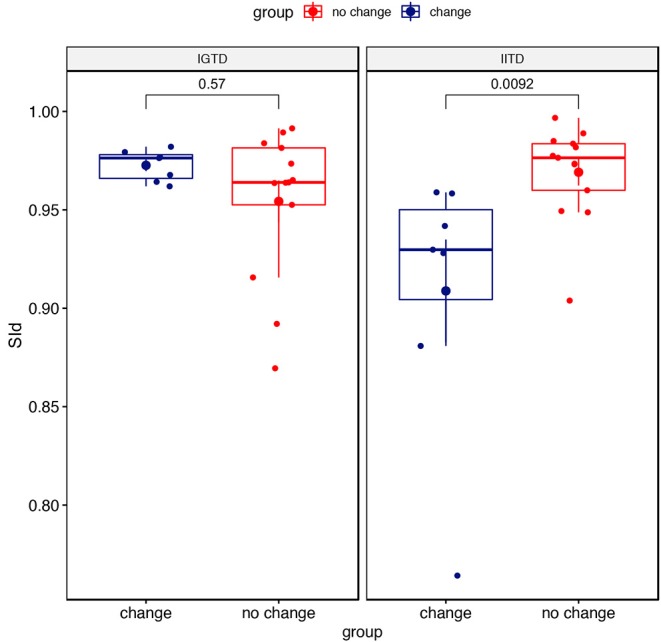
SId from the IITD and IGTD detection methods depending on the change in disease status. The EDSS was not available for one patient.

Because the SId with IITD detection was correlated with severity, it was a good candidate for use in follow-up. Therefore, we evaluated its potential for providing meaningful information for evaluating disease change. SId values with the IITD were lower for individuals with clinical changes during the 6 months in terms of EDSS, MSWS, or FIS than for those with no change on these scales (*p* = 0.0092) (Figure 5). The SId with IGTD detection did not change when comparing the two groups (*p* = 0.57).

## 4. Discussion

This personalized-template-based step detection method has three major strengths. First, it performs accurately for detecting steps from lightly to severely altered gait in individuals with pMS. Second, it is self-evaluating and is therefore reliable for clinical use. Finally, it informs on the disease severity.

The main outcome of the method lies in the excellent performance and good accuracy of inter-session IITD (reference and detection trials performed at a 6-month interval) for pMS individuals. In particular, it has potential to compensate for the lack of tools automatically detecting steps in pMS individuals with moderate to severe disease (EDSS 5.5–6.5). The performance (precision, recall, and F-measure) and accuracy (time difference in detecting IC, time difference in detecting FC, and time difference in stance duration) for HS individuals were similar to those found in the literature for step-detection algorithms based on IMUs. In particular, the mean F-measure was 1.00 (SD 0), and F-measures of 0.989–1.00 can be found for various algorithms in the literature (25, 40–44). Likewise, the mean values for ΔIC (0.015 s [SD 0.007], i.e., 1–2 samples) and ΔFC (0.019 s [SD 0.009], i.e., approximately 2 samples) lie between the range in the literature (0.008–0.040 and 0.005–0.028 s, respectively) (25, 27, 40–45). In pMS individuals, the mean F-measure was also high [0.99 (SD 0.02) for the forward intra-session prediction], both for individuals requiring a walking aid and those who could walk without aid. The algorithm was significantly less accurate than in HS participants, with mean ΔIC and ΔFC of 0.10 (SD 0.06) and 0.05 (SD 0.04) for individuals requiring a walking aid and 0.06 (SD 0.07) and 0.03 (SD 0.04) for those who could walk without aid. Nevertheless, it is still very accurate for patients who do not need a walking aid and even reached the accuracy of some algorithms used for HS individuals such as the threshold-based method from Mannini et al. (41).

We found no other algorithm compared to a gold standard (foot-switch, electronic walkway, video motion analysis, etc.) for individuals with severe disease with which to compare our results. One study (13) provided data on accuracy for severely altered steps (individuals with EDSS 6 to 6.5), but the gold standard was another step-detection method (27), which was not specifically evaluated in MS with severe disease. Still, it reported a recall lower than 80%—decreased to 0%—for individuals with an EDSS ≥6. In particular, a strength of our study was that it included both patients with and without a walking aid. Individuals with a walking aid are a real challenge for step detection (46), because instrument load and clearing can be confounded with ICs and FCs, respectively.

Contrary to Storm et al. (13), who found lower accuracy for FCs than ICs and proposed that the accuracy was due to a smoother late stance in these patients, the accuracy was higher for FCs than ICs in the present study. Spasticity or weakness might also be involved because individuals with heavy IC timing difference (sometimes >100 ms) often showed a dropped foot.

Second, the SId was correlated with performance and accuracy, both for the IITD and IGTD, in participants with pMS. Values ≥ 0.95 and 0.96 with the IITD and IGTD indicate that the detection resulted in an F-measure ≥ 0.95. Therefore, the algorithm can be self-evaluating by using this SId: it allows the clinician to be confident in detections with SId ≥ 0.95 (for IITD detection) and 0.96 (for IGTD detection). For values lower than this threshold, the clinician should consider a calibration on the GR, because the detection used was IGTD and higher performance can be expected with IITD detection or because the detection used was IITD but the individual needed a new calibration because of gait changes. In this regard, the backward inter-session prediction was found to be as high and accurate as the forward inter-session prediction. This observation allows for retrospective analysis when a new calibration on the GR is required because of a low SId or when calibration on the GR could not be done previously. As such, tests performed before this new calibration are not lost and can still be detected retrospectively.

Third, the SId computed with the IITD proved useful for evaluating disease severity. Indeed, it showed moderate linear correlation with the EDSS and its pyramidal and sensitive subscores, with a low SId suggesting more severe disease.

What is more, the SId with the IITD can be used as a follow-up biomarker because low SId indicates changes in gait. Indeed, the SId with the IITD was lower in individuals with different clinical characteristics at M6 than M0 in the EDSS (or one of the FS scores), MSWS, or FIS. Therefore, the SId can be a red flag indicating potential degradation and could be used as a biomarker of disease status in this population. The present study only considers a time interval of 6 months, which is representative of two specialized neurological consultations for pMS patients in real life. Out of the 22 patients, eight had experienced a clinically meaningful change (around 36%). It is likely that a study over a longer time period could provide more insights into the performance aspects of the proposed method.

The study has some limitations. First, the accuracy for detecting IC was very low in the WA-pMS group (some hundreds of milliseconds). This finding might be explained by a high sensitivity to peculiar steps in the detection trial. Indeed, in looking more thoroughly into the details of the library of templates for each patient, the error was often due to the presence of small steps extracted from the detection trial. These “outlier templates” were often inserted in the detection signal, at the true position of the FC, but with a great resulting difference in IC timing. For instance, one inter-session detection from one individual from the WA-pMS group showed a very high ΔIC value (mean ΔIC 0.23 ms) but high F-measure (0.99). Removing templates with lengths below the 10th percentile decreased the timing difference to a ΔIC value of 0.12 ms but also decreased the F-measure (0.98). In these cases of severely altered gait, there is a trade-off between performance and accuracy. Second, the relatively small sample size of our dataset implies a chance of overly optimistic estimates of performance in this data-driven selection of cutoff values. Nevertheless, the risk was decreased with the use of Monte Carlo cross-validation nested by four-fold cross-validation (47). Second, with the IITD, one measure on the GaitRite® system is, for now, necessary at least every 6 months. Further evaluations are needed to evaluate whether this calibration can be spaced out. In particular, we can imagine resorting to a new calibration only when the SId is below the threshold of 0.95 that we found predictive of decreased performance (F-measure ≤ 0.95). Because backward prediction had similar performance to forward prediction, a new measure on the electronic walkway system could be organized retrospectively. Even more, the IGTD detection, with lower but still high performance, could be used first. The SId could then be used to test whether the IGTD detection was performant and, if so, no calibration on the GaitRite® would be needed. This article actually proposes two methods: the first is motor-task specific, while the second, which is to be used mainly when the latter lacks sensitivity, is performance-specific. As such, for healthy subjects and some pMS, no reference data is needed. The library of steps we built from a set of the subjects performs well for detecting most steps. However, some subjects from the pMS group have highly altered gait, which cannot be recognized using this library. As a consequence, these subjects will probably need reference data. Third, we did not perform test-retest with different assessors. Hence, the sensor placement could have an impact on the results. However, a previous study with the exact same protocol (10) showed that the effect was marginal on the recorded signals due to the use of Pearson coefficient correlation, which renormalizes the templates and signals before matching. Also, the present study takes into account this phenomenon, since sensor placements at M0 and M6 are not necessarily exactly the same and nevertheless allow good performances to be obtained. Therefore, we believe that it is more a question of training for the operator and that this effect is marginal compared to the possible modifications of the gait patterns. Finally, it should be noted that the present article only deals with rectilinear walking in laboratory settings: scores can be different in real-life settings.

## 5. Conclusion

This personalized step-detection algorithm method shows high performance for detecting steps due to severely altered gait in individuals with pMS. The method can be self-evaluating with the SId: with SId ≥ 0.95, the clinician can be confident in the step detection, or with an SId lower than this threshold, he/she can perform a new test. Also, the SId can be used as a biomarker of disease state and complements the use of other scales for individual follow-up. We propose two detection methods. (1) The IGTD is less constraining because it does not require preliminary calibration with the GaitRite; however, it is less performant in step detection, and the SId is not correlated with disease state and does not reflect a change in disease status. (2) Conversely, the IITD is more constraining because it requires the use of a gold standard at least once to extract gait events, but it performs very well in detecting all types of steps and can be used as a biomarker of disease status and a red flag of disease change in a 6-month follow-up.

## Data Availability Statement

The datasets generated for this study are available on request to the corresponding author.

## Ethics Statement

The studies involving human participants were reviewed and approved by Protection des Personnes Nord Ouest III (ID RCB: 2017-A01538-45). The patients/participants provided their written informed consent to participate in this study.

## Author Contributions

AV-J conceived the study and the statistical analysis and wrote the manuscript. LO conceived the study and participated in the analysis. AM conceived the study, acquired the data, and participated in the analysis. FQ conceived the study. SE, MD, and EL acquired the data and participated in the analysis. DR conceived the study and wrote the manuscript. PV conceived the study and wrote the manuscript. All authors contributed to refining the study protocol and approved the final manuscript.

## Conflict of Interest

The authors declare that the research was conducted in the absence of any commercial or financial relationships that could be construed as a potential conflict of interest.
